# Mt10 Vaccine Protects Diversity Outbred Mice from CVB3 Infection by Producing Virus-Specific Neutralizing Antibodies and Diverse Antibody Isotypes

**DOI:** 10.3390/vaccines12030266

**Published:** 2024-03-04

**Authors:** Mahima T. Rasquinha, Kiruthiga Mone, Meghna Sur, Ninaad Lasrado, Chandirasegaran Massilamany, Stephen D. Kachman, David Steffen, Jay Reddy

**Affiliations:** 1 School of Veterinary Medicine and Biomedical Sciences, University of Nebraska-Lincoln, Lincoln, NE 68583, USA; mrasquinha2@huskers.unl.edu (M.T.R.); kmone2@huskers.unl.edu (K.M.); msur2@huskers.unl.edu (M.S.); dsteffen1@unl.edu (D.S.); 2 Center for Virology and Vaccine Research, Beth Israel Deaconess Medical Center, Harvard Medical School, Boston, MA 02115, USA; nlasrado@bidmc.harvard.edu; 3 CRISPR Therapeutics, Boston, MA 02127, USA; mchandirasegaran@gmail.com; 4 Department of Statistics, University of Nebraska-Lincoln, Lincoln, NE 68583, USA; steve.kachman@unl.edu

**Keywords:** live attenuated vaccine, coxsackievirus B3, diversity outbred mice, pancreatitis

## Abstract

Group B coxsackieviruses (CVBs) cause a wide range of diseases in humans, but no vaccines are currently available to prevent these infections. Previously, we had demonstrated that a live attenuated CVB3 vaccine virus, Mutant 10 (Mt10), offers protection against multiple CVB serotypes as evaluated in various inbred mouse strains; however, the applicability of these findings to the outbred human population remains uncertain. To address this issue, we used Diversity Outbred (DO) mice, whose genome is derived from eight inbred mouse strains that may capture the level of genetic diversity of the outbred human population. To determine the efficacy of the Mt10 vaccine, we established the CVB3 infection model in the DO mice. We noted that CVB3 infection resulted mainly in pancreatitis, although viral RNA was detected in both the pancreas and heart. Histologically, the pancreatic lesions comprised of necrosis, post-necrotic atrophy, and lymphocyte infiltration. In evaluating the efficacy of the Mt10 vaccine, both male and female DO mice were completely protected in challenge studies with CVB3, and viral RNA was not detected in the heart or pancreas. Likewise, vaccine recipients of both sexes showed significant levels of virus-neutralizing antibodies. Furthermore, by using the CVB3 viral protein 1, virus-reactive antibodies were found to be diverse in the order of IgG2c, followed by IgG2a, IgG2b/IgG3, and IgG1. Together, the data suggest that the Mt10 vaccine virus can offer protection against CVB infections that may have translational significance.

## 1. Introduction

Coxsackieviruses belonging to the *Enterovirus* genus of the *Picornaviridae* family are classified under *Enterovirus A* and *Enterovirus B* species [1,2]. *Enterovirus A* species consisting of Coxsackievirus A serotypes primarily cause diseases associated with the skin, mucous membranes, and musculoskeletal systems, such as Hand, Foot, and Mouth disease [3,4,5]. The Coxsackievirus B (CVB) viruses belonging to the *Enterovirus B* species exist in six serotypes and can cause systemic and severe illnesses such as myocarditis (CVB3 and CVB5), pancreatitis (CVB1 and CVB4), and meningitis (CVB2 and CVB5) with varied severities in those affected [4,6,7,8,9]. Chronically affected individuals can develop dilated cardiomyopathy and type 1 diabetes (T1D), as shown with CVB3 and CVB1/CVB4, respectively [10,11,12,13,14]. Although all these infections are preventable, no vaccines against them are currently available, and it is not practical to develop serotype-specific vaccines. This complexity presents a challenge to determine which CVB serotypes should be considered in the vaccine design.

In our efforts to develop a vaccine, we created a live-attenuated CVB3 vaccine strain, designated Mt10, bearing a mutation in the coxsackievirus and adenovirus receptor (CAR)-binding region of the CVB3 viral canyon [15]. We demonstrated that the Mt10 vaccine provides protection against both homologous (CVB3) and heterologous (CVB1 and CVB4) serotypes in myocarditis-susceptible A/J mice by inducing virus-specific neutralizing antibodies (nAbs) and T cell responses [15,16]. More recently, we showed that the Mt10 vaccine could prevent CVB4-accelerated T1D in the NOD/ShiLtJ (hereafter called NOD) mouse model by inducing cross-protective immune responses [17]. While these inbred mouse strains are useful models of vaccine responses, their translational relevance is questionable because testing in one inbred mouse strain is genetically akin to testing in a single person because of their limited genetic diversity. Inbred mouse models are more likely to reveal idiosyncratic features of the strain, rather than broadly translational results. Thus, animal models that capture an outbred human population’s genetic and phenotypic diversity are expected to have greater translational relevance.

To address the absence of genetic diversity in inbred mouse models, we sought to evaluate the efficacy of the Mt10 vaccine in Diversity Outbred (DO) mice. Essentially, DO mice are a mouse population derived from eight founder strains. These include three classical laboratory strains (A/J, C57Bl/6J, and 129S1/SvImJ) that have been used extensively in biological research, including knockout models [18,19,20]; two mouse strains for common human diseases, NOD for T1D and NZO/HlLtJ for obesity [21,22]; and three wild-derived mouse strains (CAST/EiJ, PWK/PhJ, and WSB/EiJ) [18,19,23]. Furthermore, all eight founder strains capture single nucleotide polymorphisms (SNPs) and insertions/deletions at approximately four times the number of SNPs found in inbred mice [24]. Therefore, the DO mice carry a level of genetic diversity comparable with the human population, thus offering enormous genetic heterozygosity and allelic diversity to investigate vaccine responses that may have translational significance [25]. In this study, after establishing the CVB3 infection model, we evaluated the efficacy of Mt10, leading us to note that the vaccine provides complete protection in challenge studies with CVB3. The vaccine responses were associated with the production of nAbs and diverse immunoglobulin (Ig) G isotypes that may have translational significance. This is particularly important because most people are not immune-compromised and DO mice represent a genetically diverse population that are immune competent.

## 2. Materials and Methods

### 2.1. Mice

Five-week-old male and female DO mice were procured from the Jackson Laboratory (Bar Harbor, ME, USA). Mice were maintained according to the institutional guidelines of the University of Nebraska-Lincoln, Lincoln, NE, USA, and approved for animal studies by the university’s Institutional Animal Care and Use Committee (IACUC). Mice were housed up to five per cage in filter-top cages assembled with closed air circulation. Cages containing the chow diet and waterers were changed biweekly until the end of the experiment. The animals had ad libitum access to food and water during the entire study period. Rooms housing the mice had sentinel cages to monitor the health of the experimental colony and avoid cross-contamination between cages. Of note, male DO mice tended to be aggressive/jumpy compared to females, often biting tails while fighting, leading to open wounds that required us to randomly remove four mice from different study groups. As a result, some of the male DO mice needed to be housed singly. Regardless of sex, DO mice were found to grind food pellets from hoppers frequently. Infection and vaccine studies were performed according to biosafety level 2 guidelines, and euthanasia was performed using carbon dioxide (CO_2_) as recommended by the Panel on Euthanasia of the American Veterinary Medical Association [26].

### 2.2. Proteins

The full-length CVB3 viral protein 1 (VP1) was expressed in a bacterial expression system and purified to remove endotoxins (GenScript, Piscataway, NJ, USA). Lyophilized keyhole limpet hemocyanin (KLH) protein was procured commercially (Sigma-Aldrich, St. Louis, MO, USA) and dissolved in deionized water. CVB3 VP1 and KLH were aliquoted and stored at −80 °C and −20 °C, respectively, until further use.

### 2.3. Infection Studies

CVB3 Nancy and CVB4-E2 strains were propagated and titrated as described previously [15,16]. For infection studies, the virus stock was diluted in 1 × phosphate-buffered saline (PBS) and administered intraperitoneally (i.p.) in various formats: (a) 1 × 10^3^ tissue culture infective dose 50 (TCID_50_) CVB3 + 1 × 10^3^ TCID_50_ CVB4/200 µL; (b) 2.5 × 10^3^ TCID_50_ CVB3 + 2.5 × 10^3^ TCID_50_ CVB4/200 µL; (c) 1 × 10^4^ TCID_50_ CVB3/200 µL; (d) 2 × 10^4^ TCID_50_ CVB3/200 µL; (e) 1 × 10^6^ TCID_50_ CVB3/500 µL. Each dose was administered to 5 males and 5 females. Sera were collected via the retro-orbital route on day 0. The control (uninfected) mice received only 1 ×  PBS. Animals were inspected twice daily during the first week after infection and once every 2–3 days thereafter until termination. Body weights were recorded every 2–3 days for the duration of the study. Animals were euthanized on day 21 post-infection, and sera, hearts, and pancreata were collected for in vitro experimentation.

### 2.4. Vaccination and Challenge Studies

The creation of Mt10 has been described in detail in our previous study [15]. Briefly, the Mt10 vaccine was derived from an infectious clone of CVB3 where a mutation (histidine to alanine) was introduced at position 790 in the VP1 within the viral canyon such that the virus loses its pathogenicity without losing immunogenicity. For vaccine studies, the Mt10 vaccine virus stock was propagated and titrated as described previously [15], diluted in 1 ×  PBS to contain 1 × 10^6^ TCID_50_/200 µL, and administered i.p. on days 0 and 21 (*n* = 13 mice/sex). Control mice (saline recipients; *n* = 15–17 mice/sex) received 1 ×  PBS at the same time points. On day 28, vaccinated and control mice were divided into two groups each and were challenged with 1 × 10^7^ TCID_50_ CVB3/1 mL (*n* = 6–9 mice/sex) or 1 ×  PBS (*n* = 6–9 mice/sex). Sera were collected on days 0, 21, 28, and at termination on day 49 (i.e., day 21 post-infection) and hearts and pancreata were harvested.

### 2.5. Histopathology

For fixation, hearts and pancreata were immersed in 10% phosphate-buffered formalin. Tissues were trimmed, processed overnight, and embedded in paraffin before they were sliced into 5 µm thick cross-sections and stained with hematoxylin and eosin (H and E). The sections were examined by a board-certified pathologist blinded to treatment. Heart sections were evaluated for inflammatory foci, mineralization, and fibrosis [27,28]. Pancreatic sections were examined for atrophy (defined as loss of acinar cells and collapse of parenchyma leaving only ducts, islets, and adipose tissue), lymphocyte infiltration, and necrosis [15,20]. The severity of pancreatic damage was assessed by determining the percentage of tissue section affected in a randomly selected portion of the pancreas.

### 2.6. RNA Isolation and Real-Time Quantitative Polymerase Chain Reaction (qPCR)

Approximately 20–30 mg of heart and pancreatic tissue harvested from various groups were snap-frozen in liquid nitrogen and stored at −80 °C. For RNA isolation, tissues were transferred to a lysis buffer containing β-mercaptoethanol and homogenized with a FastPrep-24™ system as recommended (Lysing Matrix D 1.4-mm ceramic beads; MP Biomedicals, Irvine, CA, USA). RNA was isolated using the PureLink™ RNA Mini Kit (Thermo Fisher Scientific, Waltham, MA, USA), eluted in RNase-free water, and quantified using the NanoDrop ND-1000 spectrophotometer (Thermo Fisher Scientific). In a single-step reaction, 50 ng of RNA was reverse-transcribed, and qPCR was performed using the iTaq™ Universal SYBR^®^ Green One-Step Kit (BioRad, Hercules, CA, USA). The real-time qPCR analysis included amplifications for the following genes using sequence-specific primers: CVB3 VP1 forward 5′GCGGTATGCTGAATGGGTATTA and reverse 5′CATAACAAGTACTCAACAGCCCT, and reference gene glyceraldehyde-3-phosphate dehydrogenase (GAPDH) forward 5′CTCCCACTCTTCCACCTTCG and reverse 5′GCCTCTCTTGCTCAGTGTCC. The CFX96 Touch Real-time PCR detection system (BioRad) was used for thermocycling, and BioRad CFX Maestro 1.0 software (version: 4.0.2325.0418) was used to measure the relative normalized gene expression [15,29,30] of CVB3 VP1.

### 2.7. Virus Neutralization Assay

Sera obtained from mice on days 0, 21, 28, and 49 were subjected to a virus neutralization test [15]. Vero cells were plated in 96-well tissue culture plates at a density of 0.25 × 10^6^ cells/mL to obtain 90–100% confluency. Samples were heat-inactivated at 56 °C for 30 min, and two-fold serial dilutions from 1:40 to 1:5120 were made. An equal volume of CVB3 containing 100 TCID_50_/mL was incubated with the serially diluted sera at 37 °C in a humidified chamber with 5% CO_2_. After incubation for 1 h, 100 µL of each dilution mixture was added in triplicates to plates containing cell monolayers and incubated at 37 °C for four days. The plates were examined for cytopathic effects (CPEs), and the highest serum dilution that demonstrated protection from CPEs was determined as the neutralization titer.

### 2.8. Determination of CVB-Reactive Antibodies

Serum samples obtained at different time points were analyzed for total Ig and antibody isotypes (IgM, IgG1, IgG2a, IgG2b, IgG2c, IgG3, IgA, and IgE) by indirect enzyme-linked immunosorbent assay (ELISA). Polystyrene 96-well microtiter plates were coated with CVB3 VP1 or an irrelevant control (KLH) at a concentration of 0.5 μg/mL in 1 ×  PBS and incubated at 4 °C overnight. Plates were washed with 1 ×  PBS containing 0.05% Tween-20 and blocked with 200 μL of assay buffer (1 ×  PBS containing 2% bovine serum albumin and 5% normal goat serum) for 1.5 h at room temperature. Serum samples diluted 1:100 were then added in duplicates and incubated at 37 °C for 1 h. After washing, horseradish peroxidase (HRP)-labeled goat anti-mouse total Ig, IgM, IgG1, IgG2a, IgG2b, IgG2c, IgG3, IgA, and IgE in assay buffer (1:500) were added as secondary antibodies (Southern Biotech, Birmingham, AL, USA). The plates were incubated at room temperature for 2 h, followed by washing and adding 100 μL of 1 ×  tetramethylbenzidine substrate solution (Rockland Immunochemicals, Pottstown, PA, USA). Reactions were stopped using 1 M phosphoric acid, and the optical density (OD) values were measured at 450 nm using an automated ELISA reader (BioTek Instruments, Winooski, VT, USA).

### 2.9. Statistical Analysis

Statistical analyses and graphs were prepared using GraphPad Prism software v8.1.0 (GraphPad Software, Inc. La Jolla, CA, USA) and RStudio 4.3.2. The Shapiro–Wilk test was used to test for normality for all datasets. Data sets pertaining to body weights, virus neutralization, antibody titers, and qPCR which were normally distributed were analyzed using parametric tests (paired t test or one-way ANOVA with Dunnet’s and Tukey’s multiple comparison tests) and datasets that did not fall under normal distribution were analyzed using non-parametric tests (Kruskal–Wallis test with Dunn’s multiple comparison test and Mann–Whitney U test). Barnard’s test was used to analyze the histological parameters using R v4.2.2 with the barnard.test function from the Barnard package.

## 3. Results

### 3.1. DO Mice Infected with CVB3 Developed Mainly Pancreatitis

We previously reported the efficacy of the Mt10 vaccine virus in various inbred mouse models of myocarditis and T1D [15,16,17]. In this report, we evaluated the vaccine responses in DO mice, given their extensive genetic diversity comparable to an outbred human population [25,31]. Prior to testing the vaccine efficacy, it was necessary to titrate the viral dose to induce infection in the DO mice. In this setting, we infected groups of male and female DO mice with three doses of CVB3 (1 × 10^4^, 2 × 10^4^, and 1 × 10^6^ TCID_50_) and harvested tissues at termination on day 21 post-infection. Clinically, no abnormalities were noted in any group and none of the animals lost body weight (Figure S1). Additionally, all the groups of animals had VP1-reactive total Ig (Figure S1). Histological analysis revealed mild mineralization and endocardial myocarditis in the females (2/5, 40%) after receiving 1 × 10^4^ TCID_50_ CVB3. Likewise, one male (1/5, 20%) had a small focus of fibrosis at 1 × 10^6^ TCID_50_. Analysis of the pancreatic sections revealed severe atrophy accompanied by interstitial lymphocytic infiltrates in the peripancreatic fat; lymphocytic pancreatitis tended to be more predominant in the females at all three doses, but significantly higher than in males at a dose of 1 × 10^6^ TCID_50_ (5/5, 100% vs. 1/5, 20%; *p* = 0.02) (Table S1). No other changes were noted, and the pancreatic islets were intact in all the infected animals. Altogether, histological evaluations indicated that the DO mice developed mainly pancreatitis of varying intensities, whereas myocarditis was a rare occurrence. Overall, because the pathological changes noted in the DO mice were independent of the virus dose, as expected due to their genetic diversity, and all the animals had anti-viral antibodies generated in response to infection, we proceeded to evaluate the efficacy of the Mt10 vaccine using the highest dose possible in challenge studies.

### 3.2. Mt-10 Vaccine Virus Offered Complete Protection against CVB3 Infection in Challenge Studies in Both Male and Female DO Mice

**Figure 1:**
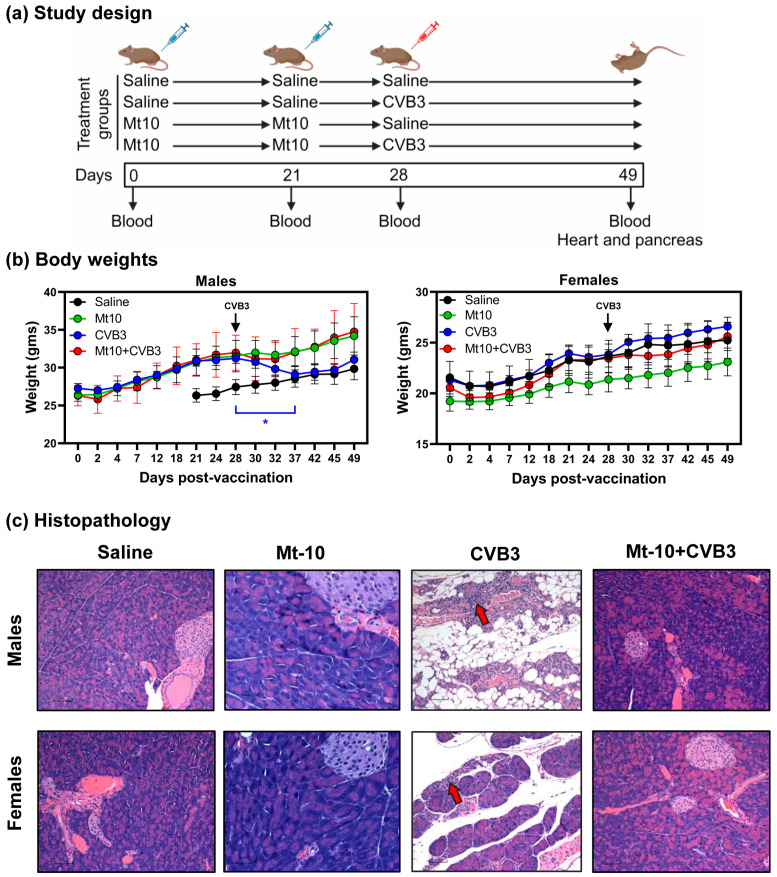
Mt10 offers protection against CVB3 infection in DO mice. (**a**) Experimental design. Groups of male and female mice were administered saline or 1 × 10^6^ TCID_50_ Mt10 vaccine on days 0 and 21. On day 28, one-half of the mice from each group were challenged with 1 × 10^7^ TCID_50_ CVB3. Serum was collected on days 0, 21, and 28 and experiments were terminated 21 days after CVB3 challenge, at which time sera and tissues were collected. (**b**) Body weights. Comparisons of body weights between treatments for males (left panels) and females (right panels) are shown. Note that the data points for male DO saline recipients are missing because the animals were aggressive, leading to severe cutaneous wounds, and they had to be euthanized according to the IACUC protocol guidelines. As a result, a fresh batch of animals was substituted. (**c**) Histopathology. Pancreata were collected from the indicated groups and processed by H and E staining to evaluate for inflammatory changes. Representative pancreatic sections for male (top panels) and female (bottom panels) mice are shown. Sections from the CVB3 group show infiltrations (red arrows). Magnification, 200×; scale bars, 60 µm. Mean ± SEM values obtained from 5 to 7 mice in each group are shown. One-way ANOVA with Dunnet’s multiple comparison test was used to determine significance between groups for body weights. * *p*  ≤  0.05. (**a**) was created with BioRender.com.

To determine the vaccine efficacy, groups of male and female DO mice were administered two doses of the Mt10 vaccine (1 × 10^6^ TCID_50_ per mouse) or saline three weeks apart, and seven days later, animals were challenged with CVB3 (1 × 10^7^ TCID_50_, the highest dose allowed by the IACUC). Blood was collected at various time points (days 21, 28 and 49) for serology, and at termination on day 21 post-challenge, tissues were harvested for histology (Figure 1a). Clinically, the vaccine-recipient, challenged, and saline groups of male and female DO mice were normal and gained body weights as expected over time. However, the DO males, but not females, in the CVB3-alone group lost body weights transitorily for a week before regaining weight thereafter (Figure 1b), and no mortalities were noted in any of the groups. Histological evaluation of heart tissues revealed no lesions in the Mt10 vaccine group. In contrast, fibrosis was detected in the hearts of two vaccine-recipient males challenged with CVB3 (2/7, 28.6%), which did not appear to be due to the vaccine or CVB3 challenge, as similar lesions were also detected in the saline groups (Table 1). However, pancreatic sections from the saline, vaccine, and vaccine/challenged groups were completely free of inflammatory changes. Expectedly, CVB3-alone groups had severe multifocal or widespread atrophy, and scattered lymphocytic infiltrates were present in both the male and female DO mice compared to the saline group (4/6, 66.7% vs. 0/9, 0%, *p* = 0.004 in males and 4/8, 50% vs. 0/9, 0%, *p* = 0.02 in females) (Table 1 and Figure 1c).

### 3.3. Viral Nucleic Acid Was Absent in the Tissues of Vaccine Recipients

**Figure 2:**
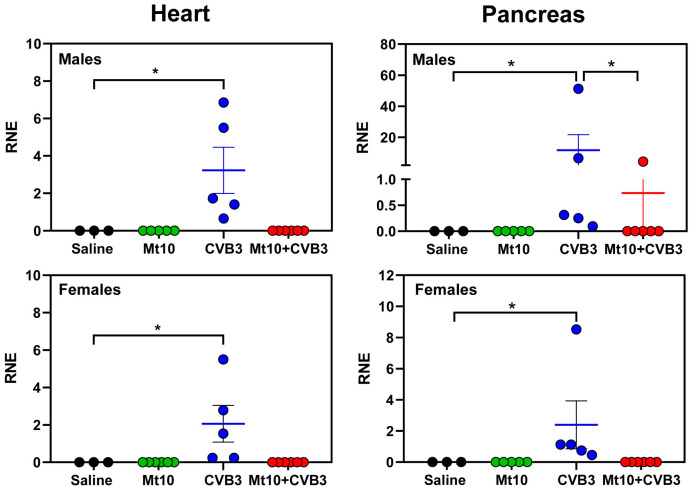
Vaccine recipients did not reveal viral RNA in the pancreas. Groups of mice were administered two doses of saline or Mt10, and 7 days later challenged with or without CVB3. Three weeks post-challenge, hearts and pancreata were collected, total RNA was extracted, and expression of CVB3 VP1 was analyzed by SYBR Green-based qPCR. The GAPDH gene was used to normalize the expression of viral RNA (∆∆Cq), and relative normalized expression (RNE) values were calculated. Mean ± SEM values representing 3–6 samples per group are shown. One-way ANOVA and the Kruskal–Wallis test were used to determine significance between groups. * *p*  ≤  0.05.

To address whether the vaccine recipients challenged with CVB3 had any residual virus in the heart and pancreas, we performed qPCR using CVB3 VP1-specific primers. Total RNA extracted from the hearts and pancreata was subjected to SYBR Green-based one-step PCR assay. The analyses revealed the presence of viral RNA in the hearts of both the males and females in the CVB3-infected animals (Figure 2, left panels). Expectedly, viral RNA was also detected in the pancreata from both sexes in the infected animals (Figure 2, right panels). While viral nucleic acids were undetectable in the hearts and pancreata from both sexes in other groups (saline, Mt10, and Mt10/CVB3) (Figure 2, left panels), viral RNA was detected in the pancreatic tissue from one male animal in the Mt10/CVB3 group (Figure 2, top right panel). Taking these findings together, the detection of CVB3 viral RNA in the pancreas can be reasoned and correlated with pancreatitis in the infected groups, but the detection of viral RNA in the heart in the absence of myocarditis may suggest that the virus can reach the heart but is unable to cause tissue damage. 

### 3.4. Vaccine Responses to Mt10 Were Associated with the Induction of nAbs, and the Virus-Reactive Antibodies Were Skewed towards IgG Isotypes

**Figure 3:**
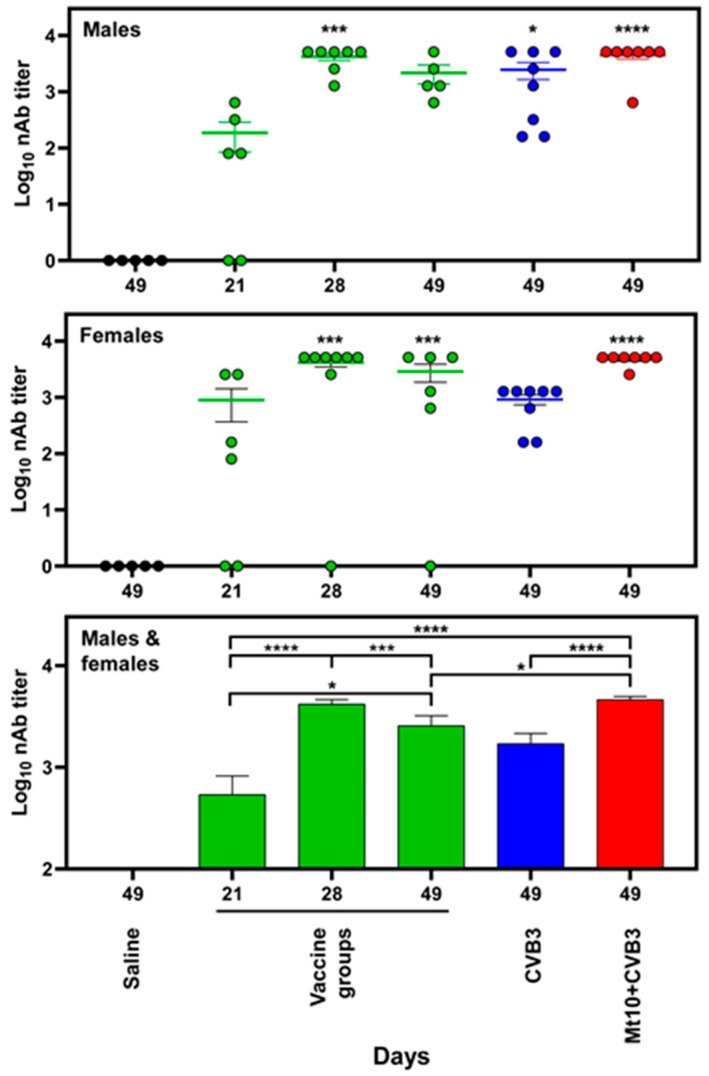
Mt10 vaccine virus induced neutralizing antibodies in both male and female DO mice. Sera were obtained from saline, vaccine alone, CVB3 alone, and Mt10/CVB3 challenged mice at the indicated time points. The serially diluted sera were incubated with CVB3, and the mixtures were transferred to 96-well plates containing Vero cells. Plates were read 4 days later to calculate the percentage neutralization based on CPEs. Mean ± SEM values obtained from 5 to 8 mice per group are shown for males (top panel), females (middle panel), and both males and females combined (bottom panel). The Kruskal–Wallis was used to determine significance between groups, and *p* values in the top and middle panels indicate differences from the saline group. * *p*  ≤  0.05, *** *p*  ≤  0.001, and **** *p*  ≤  0.0001.

To examine the nature of protective immune responses, we analyzed the production of nAbs in response to Mt10 based on CPEs [15,16,17], whereas CVB3 VP1 was used to determine the virus-reactive antibody isotypes [15,16,17]. The treatment groups included saline, Mt10 alone at different time points, CVB3 alone, and Mt10/challenged male and female DO mice. After the data were analyzed individually for each sex, the data sets of both sexes were combined and compared between groups. First, the profiles of nAbs revealed that animals receiving a single dose of Mt10 had detectable titers that were comparable between males (median titer, 80; range, 0–640) and females (median titer, 120, range, 0–2560) (Figure 3). When the animals received the second dose of Mt10, the nAb titers of both males and females were significantly elevated compared to the saline controls (Figure 3). Notably, the Mt10 vaccine groups challenged with CVB3 maintained elevated levels of nAbs in both sexes at termination (day 49), and the titers were more consistent compared with the Mt10-alone or CVB3-alone groups (Figure 3, top and middle panel). Pooling data from both sexes made it apparent that the two doses of Mt10 vaccine resulted in significantly higher levels of nAbs (median titer 5120, range, 0–5120), which showed a decline three weeks later (median titer, 1280, range, 0–5120) (day 49) but were restored in the challenged animals (median titer, 5120, range, 640–5120) that were also higher as compared to those that received one dose of the vaccine (Figure 3, bottom panel). These observations support the idea of “hybrid immunity” where vaccine responses can be boosted by infections during natural exposures, as seen with other viruses such as severe acute respiratory syndrome coronavirus 2 [32,33].

**Figure 4:**
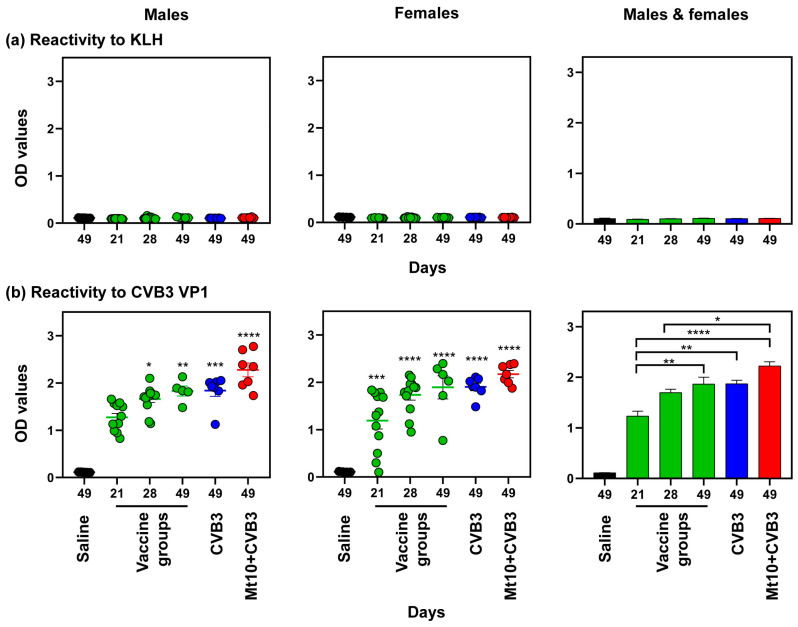
Mt10 vaccine induces CVB3 VP1-specific antibodies in DO mice. Serum samples collected from the above groups were diluted (1:100) and added in duplicates to high binding plates coated overnight with (**a**) CVB3 VP1 or (**b**) KLH (negative control). After adding HRP-conjugated goat anti-mouse total Ig detection antibody, reactions were stopped, and plates were read at 450 nm to obtain the OD values. Mean ± SEM values obtained from 5 to 8 mice for males (left panels), females (middle panels), and both males and females combined (right panels) are shown. One-way ANOVA and the Kruskal–Wallis test were used to determine significance between groups, and *p* values in left and middle panels indicate differences from the saline group. * *p*  ≤  0.05, ** *p*  ≤  0.01, *** *p*  ≤  0.001, and **** *p*  ≤  0.0001.

We next analyzed antigen-specific antibodies using KLH and VP1 as the negative control and specific antigen, respectively. Expectedly, sera obtained from the saline and treatment groups (Mt10, CVB3, and Mt10/CVB3) did not react with KLH at any time points as evaluated by total Ig concentration (Figure 4, top panels). Similar analysis for VP1 led us to detect virus-reactive total Igs in the males and females following a single dose of Mt10; the levels persisted throughout the experimental period (Figure 4, bottom left and middle panels). By pooling the data from both sexes, we noted that the VP1-reactive total Ig concentration detected after the first dose of Mt10 was elevated after the booster dose, and further increased after challenge with CVB3 (Figure 4, bottom right panel). Since the Mt10 vaccine virus-induced CVB3 VP1-reactive total Igs were comparable to, or only marginally higher than those of the CVB3 infection, we inferred that the protective responses might have been mediated by the production of IgG antibodies.

**Figure 5:**
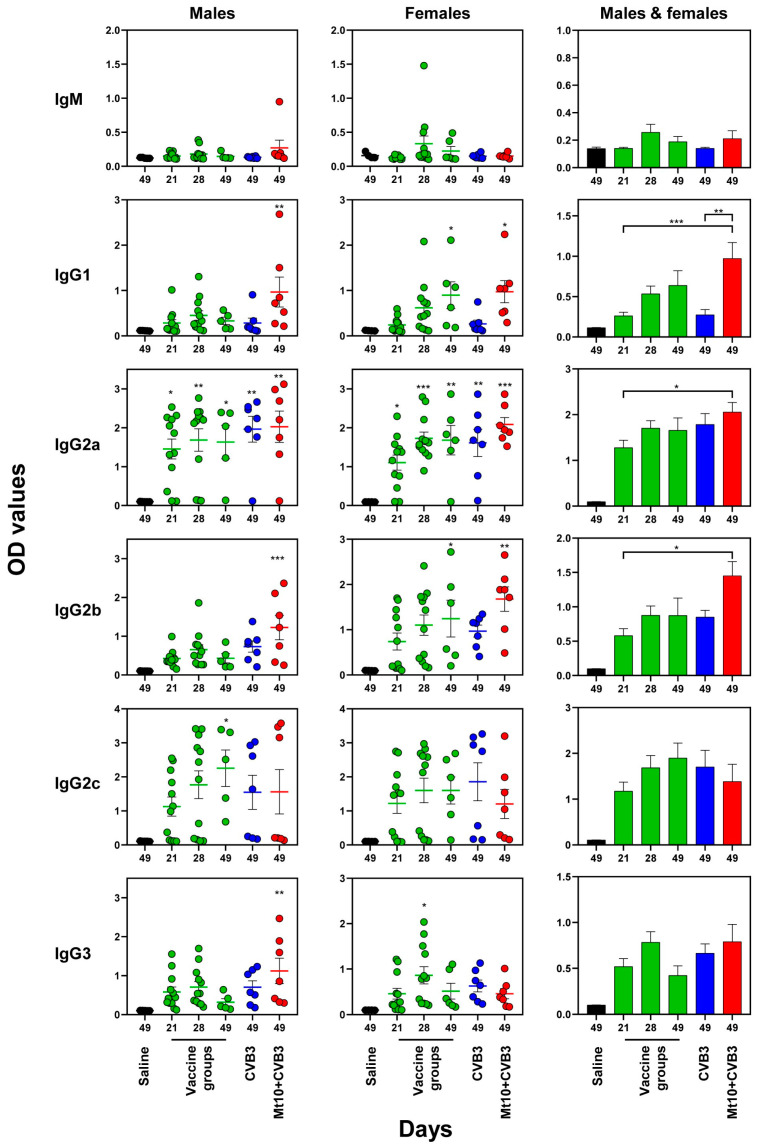
Mt10 vaccine preferentially induces IgG antibodies. High binding ELISA plates were coated with CVB3 VP1 overnight, and the diluted (1:100) serum samples from the indicated groups were added to the wells in duplicates. After adding HRP-conjugated goat anti-mouse IgM, IgG1, IgG2a, IgG2b, IgG2c, and IgG3 detection antibody, reactions were stopped, and plates were read at 450 nm to obtain the OD values. Mean ± SEM values obtained from 5 to 8 mice for males (left panels), females (middle panels), and both males and females combined (right panels) are shown. One-way ANOVA and the Kruskal–Wallis test were used to determine significance between groups, and *p* values in left and middle panels indicate differences from the saline group. * *p*  ≤  0.05, ** *p*  ≤  0.01, and *** *p*  ≤  0.001.

We investigated various antibody isotypes binding to CVB3 VP1, namely IgM, IgG1, IgG2a, IgG2b, IgG2c, and IgG3. As total Ig reactivity was lacking for KLH (Figure 4, top panels), we did not include this antigen. Likewise, we did not evaluate the production of IgA and IgE, as they were found to be absent in pilot studies. The data obtained from the males and females indicated the detection of predominantly IgG isotypes with varied levels, whereas the IgM levels were insignificant (Figure 5). While the IgG2a and IgG2c levels were detected after the first dose of Mt10 vaccine and titers remained elevated at all time points, these levels were comparable between the vaccine recipients and vaccine/challenged groups. These trends existed in both sex groups individually and in combination. In a similar analysis, elevated concentration of IgG2b was found to be relatively high in the females compared to the males, but levels in both males and females were increased in the vaccine/challenged animals. Similar patterns were noted for IgG3 and, to a lesser extent, for IgG1 in both sexes (Figure 5). Overall, increased concentrations of various IgG isotypes suggested that seroconversion had occurred in the vaccine, CVB3, and vaccine/CVB3 groups. Detection of IgG2c in only a few mice was not surprising, as IgG2c is specific to only C57BL/6J and NOD mice whose genetic contributions were also represented in the DO mice [34]. Likewise, variation in the antibody production within individual DO mice was expected given their genetic diversity. Collectively, the data suggests that the Mt10 vaccine virus offered protection against CVB3 infection in the DO mice, which was associated with the production of nAbs.

## 4. Discussion

In this report, we describe the disease phenotype of infection caused by CVB3, and the efficacy of the Mt10 vaccine in DO mice. CVB3 is mainly associated with myocarditis, and infections with CVB1 and CVB4 are often associated with T1D [11,13,14]. To prevent these infections, several vaccine approaches, such as live attenuated, killed, recombinant, and nucleic acid vaccines, have been experimentally investigated [4], but because developing a vaccine for each serotype is not a feasible option, the challenge is to develop vaccines that offer cross-protective immune responses. In that direction, we previously created the Mt10 vaccine derived from the CVB3 backbone that induces robust immune responses to the heterologous serotypes such as CVB1 and CVB4 [16,17]. We also have demonstrated the efficacy of Mt10 in challenge studies against CVB3 and CVB4 by evaluation in myocarditis-susceptible A/J mice and T1D-susceptible NOD mice [16,17]. Thus, translationally suitable preclinical models are needed since the data generated in inbred A/J or NOD mouse strains may or may not be relevant to the outbred human population. Expanding these observations in multiple inbred mouse strains is cumbersome, but the question still remains. In that direction, DO mice have been used to study the influence of genetic factors on disease susceptibility [35], physiology [36,37], autoimmunity [38], metabolism [39,40], vaccine response [41,42], behavior [43,44], and toxicity studies [45,46]. Thus, we sought to use DO mice due to their broad genetic diversity, which covers 90% of the available variation in the *Mus musculus* species [47] that are maintained as an outbred population [48]. Of note, other outbred mouse strains such as CF-1, CD-1, NMRI, and Swiss outbred mice, are derived from a single branch of the mouse family tree and are not bred for maximum diversity. The DO mice on the other hand, were derived from all seven branches of the mouse family tree and are bred to maximize the genetic diversity comparable to the human population [24,25,49,50]. Thus, observations made in the DO mice may be translationally significant because they occur across a range of genetic backgrounds rather than in a single, inbred strain.

In order to determine the efficacy of Mt10, we first established the CVB3 infection model using various doses of CVB3 in both male and female DO mice. A total of 62 DO mice, 31 of each sex, were infected at different doses. Regardless of the dose and sex, we observed pancreatitis in 58% of the animals, comprising mainly atrophy and lymphocytic infiltrations, with necrosis in isolated cases, and intact islet cells. In contrast, myocarditis was observed in only 9% of the animals with no discernable differences related to sex. In inbred mouse strains such as A/J, C57Bl/6J, and BALB/c mice, CVB infections induce severe pancreatitis [51]. As for myocarditis, while A/J and BALB/c mice develop both acute and chronic myocarditis, the disease course is restricted to the acute phase in C57Bl/6J mice [20,52]. Likewise, NOD mice, especially aged animals, develop T1D spontaneously, which can be accelerated by CVB infections, as demonstrated with mainly CVB1 and CVB4, as well as CVB3 [53,54,55]. Of note, DO mice carry a segregating allele from the myocarditis-susceptible A/J and 129S1/SvImJ mice [19], myocarditis-resistant C57Bl/6J mice, and T1D-susceptible NOD mice, in addition to the four other strains described above [54]. Thus, the combination of resistant and susceptible alleles in DO mice increases the range of response to CVBs and may more closely model the range of variation in human populations. This may mean that the increased genetic diversity increases the number of genes that could be differentially expressed and may collectively influence the occurrence of a broad range of disease phenotypes from none, to mild, to severe disease. Combining genotype and phenotype data from DO mice with broad differences in responses may lead to the identification of quantitative trait loci (QTL) controlling the disease in future studies [24]. Furthermore, the inbred mouse strains acutely develop severe pancreatitis, leading to mortalities [51,56], but none of the DO mice developed such a phenotype, and the animals were clinically normal. Such low-grade pancreatitis induced by CVBs may also reflect the phenotype of pancreatitis noted in the outbred human population, in which severe infectious pancreatitis caused by CVBs has not been routinely suspected [51,57]. Conversely, CVB1 and CVB4 infections have been shown to be associated with T1D in humans, but lack of insulitis in DO mice does not necessarily mean that they do not develop T1D, because T1D development is age-dependent due to its progressive nature even in T1D-susceptible, NOD mice [53]. Nevertheless, the lack of myocarditis in most infected animals of either sex was unexpected because viral RNA could be detected in the hearts. It may be that the virus can reach hearts but is unable to infect cardiomyocytes, the usual target cell types for CVBs, possibly due to lack of expression of receptors such as CAR and/or a decay-accelerating factor needed for viral entry [58], neither of which was investigated in this study.

After having established the CVB3 infection model, we tested the efficacy of the Mt10 vaccine in challenge studies involving the use of both male and female DO mice. In these experiments, we used the highest possible dose that could be administered—1 × 10^7^ TCID_50_ per mouse. All animals, regardless of sex, were completely protected from CVB3 infection as determined by histological analysis of the pancreata and hearts for inflammation and viral nucleic acids. Although we could not determine the lethal dose for CVB3 in challenge studies, we expect similar vaccine-induced protective effects under such conditions, and the dose we used was at least 1,000-fold higher than the usual dose we routinely use to infect susceptible A/J and C57Bl/6J mice (1 × 10^7^ vs. 1 × 10^4^ TCID_50_) [15,20]. The disease protection was accompanied by the induction of nAbs which were elevated in challenged animals when compared to the infection-alone group, suggesting that antigen (CVB3)-specific amplification of immune responses had occurred in the vaccine recipients. We have previously demonstrated that the Mt10 vaccine virus can induce cross-reactive nAbs to CVB4 and CVB1 in both A/J and NOD mice [16,17], and it is possible that DO mice could induce similar responses. However, isotyping of antibodies generated in response to the Mt10 vaccine revealed a broad range of IgG that included all isotypes reported in various strains. These include IgG2c, which is found in two of the eight founder mice (C57Bl/6J and NOD) [34], in addition to the isotypes commonly reported in various other mouse strains (IgG2a, IgG2b, IgG1, and IgG3). While detection of all these antibody isotypes was not surprising since DO mice are genetically diverse, possessing the genomes of A/J, C57Bl/6J, and NOD mice in addition to others [44], a majority of the animals in each group of males and females responded consistently, and their antibody titers were comparable. These observations suggest that the immune response genes needed to generate effective antibody responses might function independent of those that influence the disease phenotype, since only about 50% of the animals developed the disease. Additionally, due to their genetic diversity, studies in DO mice may reveal unexpected outlier phenotypes [49], but we noted no adverse events in the DO mice of either sex that received the Mt10 vaccine. Nevertheless, the finding that vaccine recipients were protected from CVB3 infection, in association with the production of nAbs, suggests that the antibodies might have played a key role in the prevention of infection. In the follow-up studies however, it would be informative to determine if the Mt10 vaccine can also protect against diseases such as T1D in DO mice, and possibly in non-human primate preclinical models.

## 5. Conclusions

Overall, we have demonstrated for the first time that DO mice can be infected by CVBs. Moreover, using CVB3 as a prototypic serotype capable of inducing both pancreatitis and myocarditis, we noted the occurrence of pancreatitis with varied disease severity in males and females with similar histological features. The DO mice, however, remained resistant to the development of myocarditis for the most part, except for a few mice (~9%) whose myocarditis may not have been due to CVB3 infection since isolated lesions were found in the saline groups. Since each DO mouse is genetically unique, it is possible that CVB3 can still induce myocarditis in DO mice, but an effort to determine this may require the use of a greater number of mice [49]. Alternatively, the use of collaborative cross (CC) mice derived from the same founder lines as DO mice may be better suited to capture the disease phenotypes. Of note, the CC mice also contain similar levels of genetic diversity but are maintained by inbreeding to keep the same genotype that may facilitate comparison of different treatment groups reproducibly. Nonetheless, the finding that the Mt10 vaccine showed remarkable efficacy in DO mice, despite being genetically diverse and outbred, indicates that the vaccine responses noted in our study may be translationally relevant to the outbred human population.

## Figures and Tables

**Table 1:** Histological analysis of hearts and pancreas in vaccinated DO mice.

Parameters	Saline		CVB3		Mt10		Mt10 + CVB3	
	Males	Females	Males	Females	Males	Females	Males	Females
Mortality	0/9 (0.0)	0/9 (0.0)	0/6 (0.0)	0/8 (0.0)	0/6 (0.0)	0/6 (0.0)	0/7 (0.0)	0/7 (0.0)
Heart								
Incidence	1/9 (11.1)	1/9 (11.1)	0/6 (0.0)	1/8 (12.5)	0/6 (0.0)	0/6 (0.0)	2/7 (28.6)	0/7 (0.0)
Myocardial lesions	1/9 (11.1)	1/9 (11.1)	0/6 (0.0)	1/8 (12.5)	0/6 (0.0)	0/6 (0.0)	2/7 (28.6)	0/7 (0.0)
Pancreas								
Incidence	0/9 (0.0)	0/9 (0.0)	4/6 (66.7) **	4/8 (50.0) *	0/6 (0.0)	0/6 (0.0)	0/7 (0.0)	0/7 (0.0)
Atrophy	0/9 (0.0)	0/9 (0.0)	4/6 (66.7) **	4/8 (50.0) *	0/6 (0.0)	0/6 (0.0)	0/7 (0.0)	0/7 (0.0)
Infiltration	0/9 (0.0)	0/9 (0.0)	4/6 (66.7) **	4/8 (50.0) *	0/6 (0.0)	0/6 (0.0)	0/7 (0.0)	0/7 (0.0)
Necrosis	0/9 (0.0)	0/9 (0.0)	0/6 (0.0)	0/8 (0.0)	0/6 (0.0)	0/6 (0.0)	0/7 (0.0)	0/7 (0.0)

() indicates percentages. * *p* ≤ 0.05, ** *p* ≤ 0.01, denotes significant differences in comparison with the saline group.

## Supplementary Materials

The following supporting information can be downloaded at: https://www.mdpi.com/article/10.3390/vaccines12030266/s1, Figure S1: Body weight changes and virus-reactive antibody responses in DO mice infected with CVB3. (a) Body weights. Mice were infected with three different doses of CVB3 as indicated and euthanized 21 days later. Animals were weighed every 2–3 days for the duration of the study. (b) Virus-reactive total Ig. Serum samples collected on days 0 and 21 were diluted (1:100) and added in duplicates to plates coated with CVB3 VP1 and incubated overnight. After adding the HRP-conjugated goat anti-mouse total Ig detection antibody, reactions were stopped, and plates were read at 450 nm to obtain the OD values. Mean ± SEM values obtained from 5 mice per group are shown. One-way ANOVA with Dunnet’s multiple comparison test was used to determine the significance between groups for body weights, and the Mann–Whitney U test was used to determine the significance for antibody responses. ** *p* ≤ 0.01; Table S1: Histological analysis of hearts and pancreas in DO mice infected with CVB3.
