# Supplementary material for: Mt10 Vaccine Protects Diversity Outbred Mice from CVB3 Infection by Producing Virus-Specific Neutralizing Antibodies and Diverse Antibody Isotypes
Source: Vaccines (Basel). 2024 Mar 4;12(3):266. doi: 10.3390/vaccines12030266 (PMC10975958; doi:10.3390/vaccines12030266)
Supplement: Supplementary file 1 [file vaccines-12-00266-s001.zip › vaccines-2856369-Table S1.pdf]

Table S1: Histological analysis of hearts and pancreas in DO mice infected with CVB3.

Parameters	1×10^4 $TCID_{50}$		2×10^4 $TCID_{50}$		1×10^6 $TCID_{50}$	
	Males	Females	Males	Females	Males	Females
Mortality	0/5 (0.0)	0/5 (0.0)	0/5 (0.0)	0/5 (0.0)	0/5 (0.0)	0/5 (0.0)
**Heart**						
Incidence	0/5 (0.0)	2/5 (40.0)	0/5 (0.0)	0/5 (0.0)	1/5 (20.0)	0/5 (0.0)
Myocardial lesions	0/5 (0.0)	2/5 (40.0)	0/5 (0.0)	0/5 (0.0)	1/5 (20.0)	0/5 (0.0)
**Pancreas**						
Incidence	4/5 (80.0)	4/5 (80.0)	1/5 (20.0)	2/5 (40.0)	2/5 (40.0)	5/5 (100.0)
Atrophy	3/5 (60.0)	3/5 (60.0)	0/5 (0.0)	2/5 (40.0)	1/5 (20.0)	5/5 (100.0) *
Infiltration	4/5 (80.0)	4/5 (80.0)	1/5 (20.0)	2/5 (40.0)	2/5 (40.0)	5/5 (100.0)
Necrosis	0/5 (0.0)	0/5 (0.0)	0/5 (0.0)	1/5 (20.0)	0/5 (0.0)	0/5 (0.0)

() indicates percentages. * $p \leq 0.05$, Denotes a significant difference in comparison with males at 1×10^6 $TCID_{50}$.
