# Supplementary figures and images for: Mt10 Vaccine Protects Diversity Outbred Mice from CVB3 Infection by Producing Virus-Specific Neutralizing Antibodies and Diverse Antibody Isotypes
Source: Vaccines (Basel). 2024 Mar 4;12(3):266. doi: 10.3390/vaccines12030266 (PMC10975958; doi:10.3390/vaccines12030266)

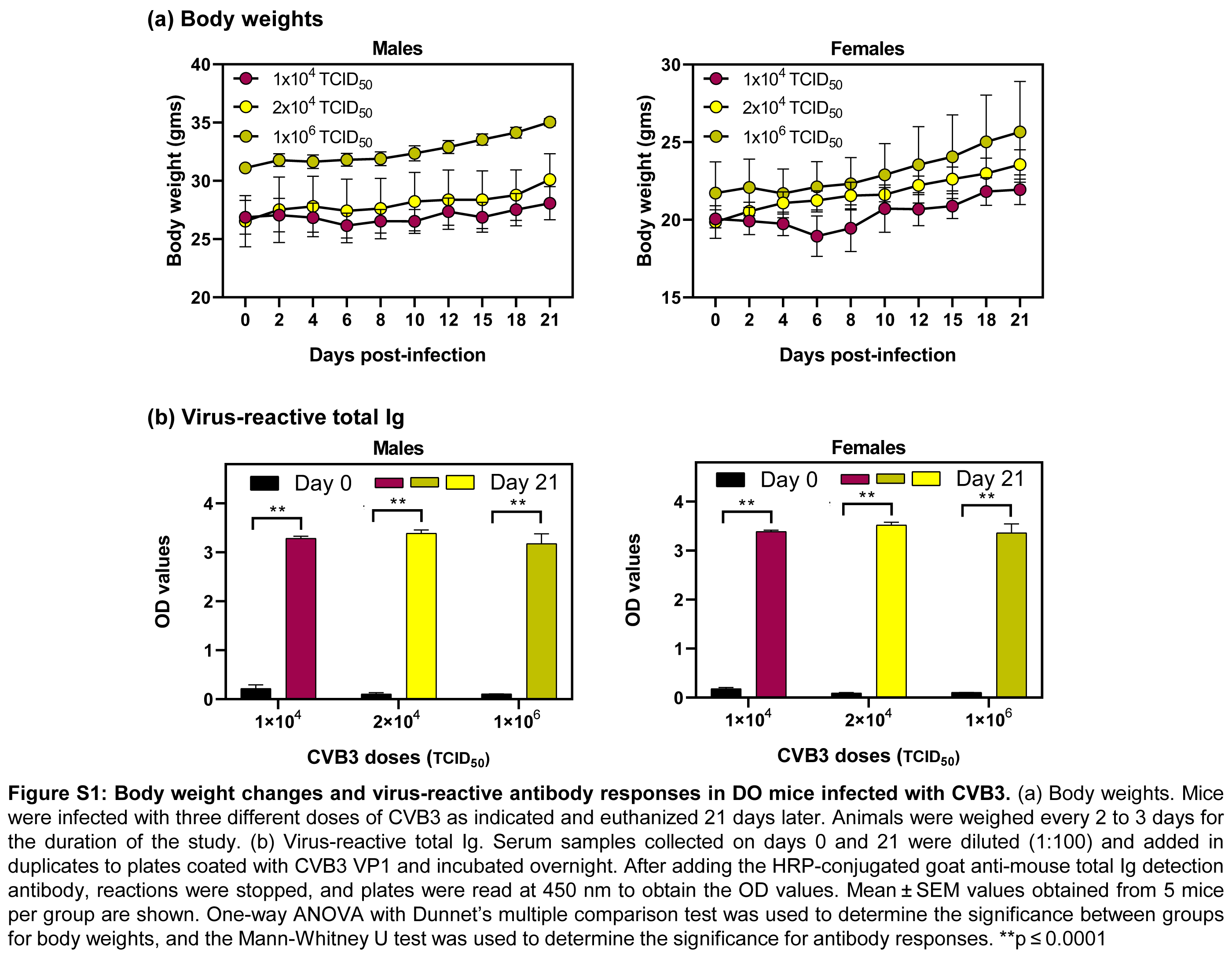

Supplement: Supplementary file 1 [file vaccines-12-00266-s001.zip › vaccines-2856369-Figure S1.tif]
